# Supplementary material for: Global Stress Detection Framework Combining a Reduced Set of HRV Features and Random Forest Model
Source: Sensors (Basel). 2023 May 31;23(11):5220. doi: 10.3390/s23115220 (PMC10255919; doi:10.3390/s23115220)
Supplement: Supplementary file 1 [file sensors-23-05220-s001.zip › sensors-2305753-supplementary.pdf]

Global Stress Detection Framework combining a reduced set of HRV features and Random Forest Model

Kamana Dahal, Brian Bogue-Jimenez, and Ana Doblas*

Department of Electrical and Computer Engineering, The University of Memphis, Memphis, TN 38152, USA
**adoblas@memphis.edu*

Abstract: This document provides supplementary information to "Global Stress Detection Framework combining a reduced set of HRV features and Random Forest Model". Here, we include Tables showing the performance metric of the trained RF model for the SWELL, WESAD, and combined dataset using the global stress framework (e.g., global training but individual testing).

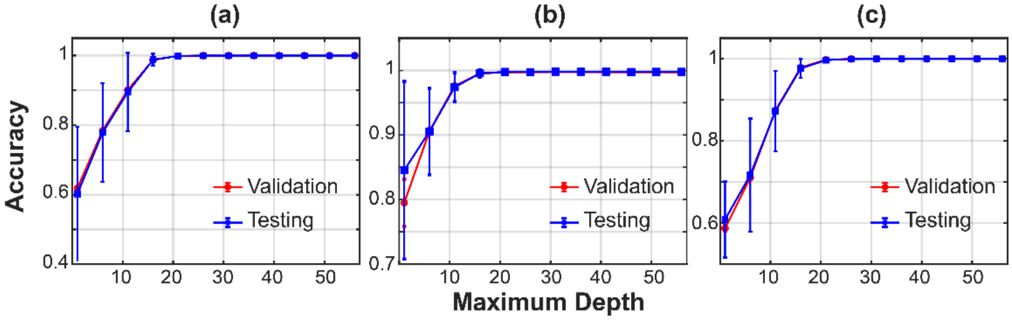

Figure S1. Hyperparameter tuning - Evaluation of the model's accuracy versus the maximum depth metric for (a) SWELL, (b) WESAD, and (c) combined datasets. The validation accuracy is the mean of the validation score over the 15 folds, and the testing accuracy is the mean value of the individual accuracy for the different subjects. The prediction power of the model in the testing dataset is not penalized for increasing the tree depth, demonstrating that our model is not overfitted.

Table S1. Performance metric of the trained RF model for the SWELL dataset using the global stress framework (e.g., global training but individual testing).

Subject id	Test samples	Accuracy	Precision	Recall	F1-score
**1**	5,754	1.00	1.00	1.00	1.00
**2**	5,819	1.00	1.00	1.00	1.00
**3**	4,805	1.00	1.00	1.00	1.00
**4**	5,832	1.00	1.00	1.00	1.00
**5**	5,415	1.00	1.00	1.00	1.00

**6**	6,267	1.00	1.00	1.00	1.00
**7**	5,272	0.99	0.99	0.99	0.99
**9**	4,783	0.99	0.99	0.98	0.99
**10**	5,904	1.00	1.00	1.00	1.00
**12**	5,904	1.00	1.00	1.00	1.00
**13**	4,824	1.00	1.00	1.00	1.00
**14**	5,904	1.00	1.00	1.00	1.00
**16**	6,451	0.99	0.99	0.99	0.99
**17**	5,648	1.00	1.00	1.00	1.00
**18**	5,581	1.00	1.00	1.00	1.00
**19**	5,976	1.00	1.00	1.00	1.00
**20**	4,428	1.00	1.00	1.00	1.00
**21**	5,004	1.00	1.00	1.00	1.00
**22**	4,581	1.00	1.00	1.00	1.00
**23**	4,047	1.00	1.00	1.00	1.00
**24**	4,824	0.99	1.00	0.99	0.99
**25**	4,475	1.00	1.00	1.00	1.00

Table S2. Performance metric of the trained RF model for the WESAD dataset using the global stress framework (e.g., global training but individual testing).

Subject_id	Test samples	Accuracy	Precision	Recall	F1-score
**2**	2,607	1.00	1.00	1.00	1.00
**3**	2,661	1.00	1.00	1.00	1.00
**4**	2,687	0.99	0.99	0.98	0.99
**5**	2,702	0.99	1.00	0.99	0.99
**6**	2,695	1.00	1.00	1.00	1.00
**7**	2,691	1.00	1.00	1.00	1.00
**8**	2,700	1.00	1.00	1.00	1.00
**9**	2,677	1.00	1.00	1.00	1.00
**10**	2,806	1.00	1.00	1.00	1.00
**11**	2,742	1.00	1.00	1.00	1.00
**13**	2,741	1.00	1.00	1.00	1.00
**14**	2,713	1.00	1.00	1.00	1.00
**15**	2,745	0.99	0.99	0.99	0.99
**16**	2,729	1.00	1.00	1.00	1.00
**17**	2,805	1.00	1.00	1.00	1.00

Table S3. Performance metric of the trained RF model for the combined (SWELL + WESAD) dataset using the global stress framework (e.g., global training but individual testing).

Subject id	Test samples	Accuracy	Precision	Recall	F1-score
1	5754	1.00	1.00	1.00	1.00
2	5819	1.00	1.00	1.00	1.00
3	4805	1.00	1.00	1.00	1.00
4	5832	1.00	1.00	1.00	1.00
5	5415	1.00	1.00	1.00	1.00
6	6267	1.00	1.00	0.99	1.00
7	5272	1.00	1.00	0.99	0.99
9	4783	0.99	0.99	0.99	0.99
10	5904	1.00	1.00	1.00	1.00
12	5904	1.00	1.00	1.00	1.00
13	4824	1.00	1.00	1.00	1.00
14	5904	1.00	1.00	1.00	1.00
16	6451	1.00	1.00	1.00	1.00
17	5648	1.00	1.00	1.00	1.00
18	5581	1.00	1.00	1.00	1.00
19	5976	1.00	1.00	1.00	1.00
20	4428	1.00	1.00	1.00	1.00
21	5004	1.00	1.00	1.00	1.00
22	4581	1.00	1.00	1.00	1.00
23	4047	1.00	1.00	1.00	1.00
24	4824	1.00	1.00	1.00	1.00
25	4475	1.00	1.00	1.00	1.00
26	2607	0.98	0.98	0.97	0.97
27	2661	1.00	1.00	0.99	1.00
28	2687	0.97	0.96	0.98	0.97
29	2702	1.00	1.00	1.00	1.00
30	2695	0.98	0.98	0.98	0.98
31	2691	0.99	0.99	1.00	0.99
32	2700	0.99	0.99	0.99	0.99
33	2677	0.98	0.98	0.96	0.97
34	2806	0.99	0.99	0.99	0.99
35	2742	1.00	0.99	1.00	1.00
36	2741	1.00	0.99	0.99	0.99
37	2713	0.99	0.99	1.00	0.99
38	2745	0.99	0.99	1.00	0.99
39	2729	0.98	0.97	0.99	0.98
40	2805	0.99	0.99	0.99	0.99

Table S4. Performance metric of the trained RF model for the SWELL dataset using the global stress framework and the eight selected features from the combined dataset.

Subject id	Test samples	Accuracy	Precision	Recall	F1-score
**1**	5754	1.00	1.00	1.00	1.00
**2**	5819	1.00	1.00	1.00	1.00
**3**	4805	1.00	1.00	1.00	1.00
**4**	5832	1.00	1.00	1.00	1.00
**5**	5415	1.00	1.00	1.00	1.00
**6**	6267	1.00	1.00	1.00	1.00
**7**	5272	1.00	1.00	1.00	1.00
**9**	4783	1.00	1.00	1.00	1.00
**10**	5904	1.00	1.00	1.00	1.00
**12**	5904	1.00	1.00	1.00	1.00
**13**	4824	1.00	1.00	1.00	1.00
**14**	5904	1.00	1.00	1.00	1.00
**16**	6451	1.00	1.00	1.00	1.00
**17**	5648	1.00	1.00	1.00	1.00
**18**	5581	1.00	1.00	1.00	1.00
**19**	5976	1.00	1.00	1.00	1.00
**20**	4428	1.00	1.00	1.00	1.00
**21**	5004	1.00	1.00	1.00	1.00
**22**	4581	1.00	1.00	1.00	1.00
**23**	4047	1.00	1.00	1.00	1.00
**24**	4824	1.00	1.00	1.00	1.00
**25**	4475	1.00	1.00	1.00	1.00

Table S5. Performance metric of the trained RF model for the WESAD dataset using the global stress framework and the eight selected features from the combined dataset.

Subject_id	Test samples	Accuracy	Precision	Recall	F1-score
**2**	2607	1.00	0.99	0.99	0.99
**3**	2661	1.00	1.00	1.00	1.00
**4**	2687	1.00	1.00	1.00	1.00
**5**	2702	1.00	1.00	1.00	1.00
**6**	2695	1.00	1.00	1.00	1.00
**7**	2691	1.00	1.00	1.00	1.00
**8**	2700	1.00	1.00	1.00	1.00
**9**	2677	1.00	1.00	0.99	1.00

**10**	2806	1.00	0.99	1.00	0.99
**11**	2742	1.00	1.00	1.00	1.00
**13**	2741	1.00	1.00	1.00	1.00
**14**	2713	1.00	1.00	1.00	1.00
**15**	2745	1.00	1.00	1.00	1.00
**16**	2729	1.00	1.00	1.00	1.00
**17**	2805	1.00	1.00	1.00	1.00
